# Emotional Training via Telerehabilitation After Surgical Treatment for Facial Palsy: Prospective, Assessor-Blinded, 2-Arm Pilot Cohort Study

**DOI:** 10.2196/79520

**Published:** 2026-04-27

**Authors:** Matteo Guidetti, Silvia Cupello, Jacopo Reali, Natale Vincenzo Maiorana, Sara Marceglia, Rossella Pagani, Federico Biglioli, Alberto Priori, Antonino Michele Previtera

**Affiliations:** 1 Department of Health Sciences, Aldo Ravelli Center for Neurotechnology and Experimental Brain Therapeutics, University of Milan, Milan, Italy; 2 Department of Health Sciences, Rehabilitation Unit, ASST-Santi Paolo e Carlo University Hospital, University of Milan, Milan, Italy; 3 Department of Health Sciences, University of Milan, Via Antonio di Rudinì 8, Milan, 20142, Italy, 39 02 50323233; 4 Department of Head and Neck, Division of Maxillofacial Surgery, San Paolo Hospital, University of Milan, Milan, Italy; 5 Department of Health Sciences, Clinical Neurology Unit, Azienda Socio-Sanitaria Territoriale Santi Paolo E Carlo, University of Milan, Milan, Italy

**Keywords:** peripheral facial nerve palsy, physiotherapy, motor rehabilitation, telerehabilitation, Sunnybrook Facial Grading System

## Abstract

**Background:**

Peripheral facial nerve palsy is a debilitating condition that may necessitate surgical intervention. Although motor rehabilitation is considered essential, the most effective approach has not yet been determined.

**Objective:**

This study aimed to evaluate the feasibility and effectiveness of emotional training, a novel telerehabilitation-based treatment, on motor, functional, and psychological outcomes in patients with unilateral facial palsy following triple innervation surgery.

**Methods:**

A prospective, assessor-blinded, 2-arm pilot cohort study was conducted at the rehabilitation unit at University Hospital San Paolo, Milan, Italy, from January to October 2024. Participants (N=16) received 1 treatment session every 2 weeks over 20 weeks, each lasting 45 minutes, according to standard clinical procedures in place at the rehabilitation unit. Participants were nonrandomly assigned to either an in-person group (n=8) or an online group (ie, telerehabilitation; n=8) based on their ability to attend in-person sessions. The primary outcomes assessed at baseline (T0) and after treatment (T1) included facial symmetry (Sunnybrook Facial Grading System; SFGS), facial disability (Facial Disability Index; FDI), and anxiety levels (Beck Anxiety Inventory).

**Results:**

Statistical analysis revealed significant improvements at T1 for both groups in the FDI social and well-being function subscale, Beck Anxiety Inventory, SFGS resting symmetry score, SFGS symmetry of voluntary movement score, SFGS composite score, SFGS with bilateral masseter contraction symmetry of voluntary movement score, and SFGS with bilateral masseter contraction composite score (*P*<.001 for all). Only the FDI physical function subscale showed a differential improvement at T1 for the in-person group treatment (ANOVA for time × treatment: *F*_1_=14.356; *P*=.002; Holm-Bonferroni post hoc test: *P*<.001). Finally, a strong positive correlation was observed between the time elapsed from surgery to rehabilitation and SFGS composite score improvement at T1 (*r*=0.94; *P*=.005).

**Conclusions:**

These results suggest that the online emotional training protocol is as feasible and effective as the in-person emotional training protocol in improving facial motor function, reducing anxiety, and enhancing facial expression spontaneity in patients who had undergone surgery for peripheral facial palsy. These findings support the validity of telerehabilitation approaches as a feasible, accessible, and sustainable alternative to conventional in-person therapy for facial nerve recovery.

## Introduction

Peripheral facial nerve palsy (FNP) is a neurological disorder that affects the muscles of one side of the face [1], leading to facial asymmetry; synkinesis; hemifacial spasm [2]; impaired eye and mouth closure; and functional deficits in daily activities, including vision, speech, and eating [3,4]. Peripheral FNP resulting from traumatic events (eg, cranial fractures) [5], oral and maxillofacial surgery [6], or neoplastic conditions (eg, acoustic neuroma) [6] often presents with a highly variable prognosis [6,7] and may require invasive intervention, such as facial reanimation [8]. Following facial reanimation surgery, physiotherapy (PT) plays a critical role in motor and functional recovery [9]. PT interventions capitalize on principles of neuroplasticity and motor learning to restore facial symmetry [10,11]; preserve proprioception, muscle tone, and trophism [12]; reduce synkinesis; and enhance both spontaneous and voluntary facial movements [13].

Triple innervation is a novel surgical protocol [14,15] that uses 3 distinct neural inputs to restore movements and functions: the masseteric and hypoglossal nerves, which serve as quantitative input (ie, facilitating voluntary facial movements, such as smiling during teeth clenching and maintaining muscle tone), and the facial nerve contralateral to the damaged one, which functions as a qualitative input (ie, supporting spontaneous facial expressions such as smiling and blinking) [14,15]. Several studies suggest that triple innervation surgery is associated with complication rates comparable to those of other established techniques [14] while demonstrating improved outcomes in terms of facial symmetry, voluntary and spontaneous movements, and overall functional recovery both at short- and long-term follow-up [14,16,17]. Given the novelty of the surgical technique, no study has assessed the effect of PT following triple innervation yet. In addition, although several PT protocols, including massage [12,18], electrotherapy [19,20], exercise therapy [10,11], and biofeedback therapy [21,22], have demonstrated beneficial effects, no clear evidence supports the superiority of any of them [13,23]. Compared to other pathologies, facial motor rehabilitation is complicated by the intrinsic link between facial movements and emotional expression. The neural circuits governing facial expressions are closely connected to systems involved in emotion recognition and social communication [24-27], suggesting that interventions targeting both motor and emotional components may be particularly relevant in FNP. Moreover, aesthetic and functional impairments often lead to psychosocial distress, reduced self-esteem, and increased anxiety [28,29], reinforcing the need for rehabilitation approaches that also address emotional and cognitive aspects of facial movement. Within the context of PT interventions, emotional training has recently been proposed as a targeted approach to address these neurophysiological and clinical characteristics and optimize rehabilitation outcomes [30]. Emotional training conceptualizes movement control as a cognitive process involving selective attention to sensory cues and task demands [31,32] and integrates emotional, cognitive, and motor components to facilitate selective muscle recruitment, retraining of emotional expressiveness, and the execution of functional exercises oriented toward daily activities. This approach has been recently introduced as a rehabilitation approach for patients after triple innervation surgery in our center [30].

Recently, telerehabilitation has been proposed as a promising rehabilitation method for patients with neurological disorders, showing results comparable to those of conventional face-to-face rehabilitation [33]. While still promising, evidence remains limited for the motor rehabilitation of peripheral FNP [34,35]. Telerehabilitation represents a hospitalization-avoidant strategy that emphasizes the delivery of home-based rehabilitation, minimizing resource requirements and reducing direct supervision needs [36-38]. This approach may serve as a particularly viable treatment option for patients with FNP [36] as it minimizes the need for social exposure and interpersonal contact, which can be especially beneficial for patients facing psychosocial burden.

In this work, we primarily aimed to assess the feasibility and motor-functional and psychological effects of a telerehabilitation-based emotional training intervention [30] in patients with peripheral FNP who underwent triple innervation surgery and compare the results with those of an in-person emotional training approach.

## Methods

### Study Design and Setting

This prospective, assessor-blinded, 2-arm pilot cohort study was conducted at the rehabilitation unit at University Hospital San Paolo (Milan, Italy). On the basis of their ability to attend in-person sessions, each participant eligible for enrollment (N=16) engaged in either in-person PT treatment (ie, in-person emotional training; n=8) or in telerehabilitation treatment (ie, online emotional training; n=8). In both groups, treatments were led by trained physiotherapists. Motor outcomes (Sunnybrook Facial Grading System [SFGS] and SFGS with bilateral masseter contraction [SFGS with MassCon]), functional outcomes (Facial Disability Index; FDI), and psychological outcomes (Beck Anxiety Inventory; BAI) were assessed at baseline (T0) and at the completion of treatment (T1). Given the exploratory pilot nature of this study, which aimed primarily to assess feasibility and generate preliminary results to inform future confirmatory trials, and considering the rarity of the medical condition targeted by the inclusion criteria, no formal a priori sample size calculation was performed. However, the final sample (N=16) is consistent with those of previous pilot investigations conducted in comparable clinical populations [39,40]. A convenience sampling strategy was adopted, enrolling all consecutive eligible patients referred to the rehabilitation unit during the predefined recruitment period (November 2023 to November 2024). In addition, a prospective observational design was adopted due to several clinical (eg, emotional training is part of postoperative rehabilitation in our department) and logistical (eg, rarity of the target population and allocation to treatment according to patients’ real-world logistical circumstances) reasons.

### Ethical Considerations

The study protocol followed the Declaration of Helsinki. Considering that emotional training is common practice for patients undergoing triple innervation surgery at the rehabilitation unit of University Hospital San Paolo, and that patients with peripheral FNP would have been treated with the same protocol after triple innervation surgery despite not participating in the study, the research was considered as an observational study. Ethical approval was obtained from the Milan Area 1 Ethics Committee, protocol number 0048450/2022. All patients gave written informed consent before participation. All data collected in this study were handled in accordance with strict privacy and confidentiality standards. Records were anonymized through the use of coded identifiers, and no personally identifiable information was retained in either the research database or the investigational application. Hard-copy materials (eg, consent forms) were securely stored in a locked cabinet in a restricted-access laboratory, while electronic data were maintained on protected institutional servers with controlled access. Access to the coded datasets was limited to authorized members of the research team. Participants were not provided with financial compensation for their involvement in the study.

### Population

This study was conducted between January and October 2024. Participants were recruited from the hospital community based on the following inclusion criteria: (1) age between 18 and 75 years; (2) presence of unilateral facial palsy, confirmed through clinical history and anamnesis; (3) prior treatment with triple innervation surgery (as described in the work by Biglioli et al [14]); (4) a House-Brackmann scale grade of III or higher; (5) ability to understand and perform the tasks; and (6) capacity to provide informed consent. Exclusion criteria were (1) a diagnosis of major neurological, neuropsychological, or psychiatric disorders, confirmed through clinical history and anamnesis; (2) previous treatments for facial palsy other than triple innervation surgery [14]; and (3) changes in pharmacological therapy within 3 months prior to enrollment. Patients were instructed to maintain stable medication regimens throughout the study period and refrain from participating in additional rehabilitation programs.

### Treatments

#### Overview

All patients received the standard 5-month emotional training rehabilitation program [30]. The treatment sessions lasted 50 minutes and were conducted once every 2 weeks over a 20-week period (total number of sessions=10; see Multimedia Appendix 1 for a structured session plan). The primary objective of both the in-person and online groups was to enhance the quality and symmetry of facial muscle movements, initially facilitated through teeth clenching, and progressively dissociating these movements from masseter activation [41-43]. This approach was chosen because one of the primary rehabilitative goals for patients who have undergone triple innervation surgery is to improve voluntary contraction of the mimic muscles by gradually decoupling them from masseter activation through repeated exercises and the progressive automation of movements [41-43]. According to the principles of triple innervation surgery [14], the masseteric nerve, which stimulates the masseter muscles, serves as the principal donor nerve for activating the facial nerve and, consequently, the facial mimic muscles. Therefore, postsurgical facial muscle contractions activate the masseter muscles. The progress of each patient was closely monitored by physiotherapists, who adjusted the complexity of exercises in response to individual progress. As facial movements improved, progressively more advanced exercises were introduced to stimulate emotional expression and functional facial movements in increasingly complex real-world contexts, providing continuous feedback to optimize movement patterns. Through consistent repetition and progressive automation, the secondary objective was to restore the functionality and spontaneity of facial expressions, enhancing facial perception and promoting cortical reorganization. As suggested by previous research [44], exercises were tailored to each patient’s residual motor capabilities, functional limitations, and social challenges. The physiotherapists delivering the treatment underwent extensive training prior to its initiation and had prior experience in the rehabilitation of peripheral FNP.

#### In-Person Emotional Training Protocol

Before initiating the emotional training protocol, tissue flexibility in the impaired masseter, medial and lateral pterygoid, zygomaticus major, orbicularis oculi, and orbicularis oris muscles was improved using manual therapy techniques [45] such as internal and external stretching techniques and transverse massage [45,46]. This preliminary phase was necessary due to the presentation typically observed in soft tissues following facial palsy surgery [47].

As previously reported [30], emotional training comprises 3 phases. The first phase introduces exercises designed to increase sensory awareness, promote appropriate and selective muscle recruitment, and integrate both sensory perception and motor function [32]. Patients were asked to perform tactile and pressure perception exercises, identifying sensations at key points on the face (eg, the corners of the mouth and the eyebrows) [32]. When movement abilities allowed it, facial exercises were paired with the activation of the masseter muscle focusing on 2 movements of functional importance: eye closure and smiling (with either closed or open lips). Otherwise, patients were asked to perform motor imagery exercises, which have been shown to activate the same cortical and subcortical areas as during actual movement execution [48,49]. Patients mentally simulated facial movements focusing on the simulated sensations and were then asked to perform them and compare the simulated and actual sensations. As facial movements improved, exercises progressively intensified, with patients being asked to close their eyes or smile at different intensities of teeth clenching or to self-correct their movements according to their perceived facial symmetry.

The second phase introduces exercises to improve the spontaneity of emotional expression. Patients were asked to perform facial expressions corresponding to emotions defined by Osgood [50] for nonverbal communication (happiness, sadness, interest, disgust, fear, anger, and surprise) elicited through visual (eg, viewing triggering images or scenarios), taste (eg, tasting sour food), or memory (eg, recalling triggering experiences) stimuli. As facial movements improved, patients were asked to switch between emotional expressions (eg, from fear to anger).

The last phase of the protocol focuses on functional exercises aimed at restoring the ability to perform daily activities (eg, communicating effectively or drinking from a glass) using facial muscles. These exercises included, for example, activities to improve word articulation (eg, reading aloud a text or words with challenging sounds such as labial consonants [eg, “b” and “p”]) or mastication (eg, chewing inside both cheeks or transferring food or liquid from one side to the other).

#### Online Emotional Training Protocol

Participants in the online treatment group received the same intervention protocol as those in the in-person treatment group in terms of the type, nature, frequency, and objectives of the exercises; however, the intervention was delivered remotely via video call, enabling the physiotherapist to directly monitor exercise correctness, safety, and adherence; provide immediate feedback; and adjust the intervention as needed. No session was recorded as supervision and feedback were provided live during each video call and adherence was tracked through supervised session attendance as the intervention did not include unsupervised exercises requiring additional logging or diaries. Due to the absence of in-person interaction with the physiotherapist, patients were initially trained during the assessment phase to perform manual relaxation techniques and facial exercises targeting eye closure and smiling with masseter muscle activation. These exercises were performed under the physiotherapist’s virtual supervision, where the patient was guided to repeat the maneuvers, ensuring correct execution and understanding. Furthermore, throughout the treatment sessions, exercises were designed to encourage patients to focus attentively on the sensory feedback from their facial movements, with continuous feedback provided to assess progress. No further modifications were made to the in-person emotional training protocol compared to the online emotional training protocol.

### Outcomes

SFGS, SFGS with MassCon, FDI, and BAI were administered both at baseline (T0) and the posttreatment time point (T1) by a single trained physiotherapist who was blinded to treatment group allocation but not to time points.

The SFGS is a widely used clinical scale designed to evaluate facial asymmetries and the presence of synkinesis comparing the paretic side of the face with the nonparetic side [51]. The scale consists of 3 components: the first (resting symmetry) involves assessing symmetry at rest, including the eye, cheek (nasolabial fold), and mouth; the second (symmetry of voluntary movement) rates symmetry during facial expressions (ie, during 5 standard facial expressions); and the third (synkinesis) grades the severity of synkinesis during these movements. Each component is assigned a score, and the weighted sum of these components generates a composite score ranging from 0 to 100, with a lower score indicating greater severity of facial palsy [51].

For this study, a modified version of the SFGS was used in which participants were instructed to perform the second component (symmetry of voluntary movement) while clenching their jaw (ie, activating the masseter muscles).

The FDI is a 10-item self-report questionnaire assessing 2 domains of facial disability: physical and psychosocial function [52]. It includes 2 subscales—one for physical function and the other for social and well-being function—each comprising 2 items. The scores for each subscale are converted to a 100-point scale, where a score of 100 denotes no impairment in function [52].

The BAI is a psychometrically validated, self-reported, 21-item scale commonly used to measure symptoms of anxiety [53]. The score for each item is summed, yielding a total score ranging from 0 to 63, categorized as minimal anxiety (0-7), mild anxiety (8-15), moderate anxiety (16-25), and severe anxiety (26-63) [53].

### Statistical Analysis

Normal distributions of the dependent variables were assessed using the Shapiro-Wilk test for normality [54]. As all variables passed the test (*P*>.05), parametric statistical analysis was applied. To evaluate the effects of time, treatment, and the interaction between time and treatment, changes in the outcomes at each time point were examined using a 2-way repeated-measure ANOVA with treatment as the between-subject factor. Post hoc analysis was performed using the Holm-Bonferroni correction. Finally, the Pearson correlation coefficient was calculated to explore relationships among age, time from onset of facial palsy to surgery, time from surgery to rehabilitation, time from onset of facial palsy to rehabilitation, and the change in outcome scores (∆; calculated as ∆ score = T1 score − T0 score). A *P* value of less than .05 was considered statistically significant for all analyses. Data were analyzed using JASP (version 0.16.3) for Windows (JASP Team).

## Results

**Figure 1.**
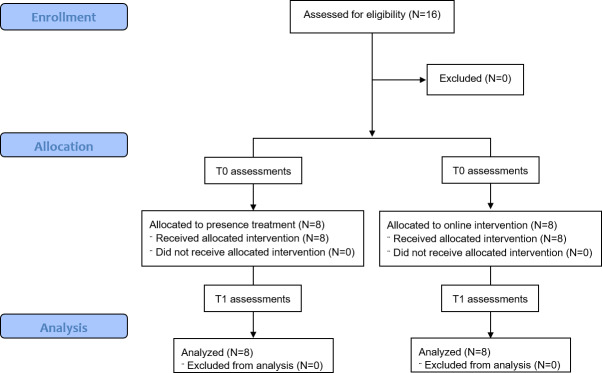
Flowchart of the study design. T0: baseline; T1: posttreatment time point.

Baseline characteristics of the sample are shown in Table 1 (see Figure 1 for the CONSORT [Consolidated Standards of Reporting Trials] flow diagram). At baseline, there were no significant differences in demographic variables or clinical assessments between the 2 treatment groups (*P*>.05 for all the analyses).

**Table 1.** Sample characteristics (N=16).

	Overall	in-person (n=8)	Online (n=8)	*P* value^a^
Sex (female), n (%)	10 (62.5)	4 (50.0)	6 (75.0)	.30
Age (y), mean (SD)	53.93 (16.63)	53.12 (18.30)	54.75 (16.02)	.88
Side, n (%)				.61
Right	7 (43.8)	3 (37.5)	4 (50.0)	
Left	9 (56.3)	5 (62.5)	4 (50.0)	
Cause, n (%)				.19
Neurinoma	12 (75.0)	5 (62.5)	7 (87.5)	
Meningioma	1 (6.3)	1 (12.5)	0 (0)	
Angioma	1 (6.3)	0 (0)	1 (12.5)	
Trauma	1 (6.3)	1 (12.5)	0 (0)	
Surgery	1 (6.3)	1 (12.5)	0 (0)	
Onset to surgery (mo), mean (SD)	21.04 (13.25)	25.70 (14.34)	11.74 (0.9)	.09
Surgery to rehabilitation (mo), mean (SD)	10.54 (4.5)	10.4 (5)	10.82 (5.16)	.52
Onset to rehabilitation (mo), mean (SD)	31.57 (13.49)	36.08 (14.7)	23 (4.27)	.13
SFGS^b^ (resting symmetry score: 0-20; symmetry of voluntary movement score: 20-100; synkinesis score: 0-15; and composite score: 0-100), mean (SD)				
Resting symmetry score	12.19 (3.15)	11.25 (3.54)	13.13 (2.59)	.29
Symmetry of voluntary movement score	45.75 (12.39)	46 (14.81)	45.5 (10.46)	.99
Synkinesis score	1.38 (1.86)	1.38 (2.13)	1.38 (1.69)	.96
Composite score (%)	32.2 (13.2)	33.4 (15.1)	31 (11.8)	.79
SFGS with MassCon^c^ (resting symmetry score: 0-20; symmetry of voluntary movement score: 20-100; synkinesis score: 0-15; composite score: 0-100), mean (SD)				
Symmetry of voluntary movement score	61.00 (10.78)	61.5 (12.64)	60.5 (9.42)	.83
Composite score (%)	47.3 (11.7)	48.5 (13.6)	46.1 (10.2)	.71
FDI^d^, mean (SD)				
Physical function subscale (range 0-100)	53.75 (22.32)	45 (18.51)	62.5 (23.45)	.11
Social and well-being function subscale (range 0-100)	58.00 (25.42)	52 (26.01)	64 (25.02)	.46
BAI^e^ total score (0-63), mean (SD)	16.81 (12.44)	20.13 (15.11)	13.5 (8.85)	.52

a Mann-Whitney *U* test (continuous variables) or chi-square test (categorical variables); in-person vs online group.

b SFGS: Sunnybrook Facial Grading System.

c MassCon: bilateral masseter contraction.

d FDI: Facial Disability Index.

e BAI: Beck Anxiety Inventory.

**Figure 2.**
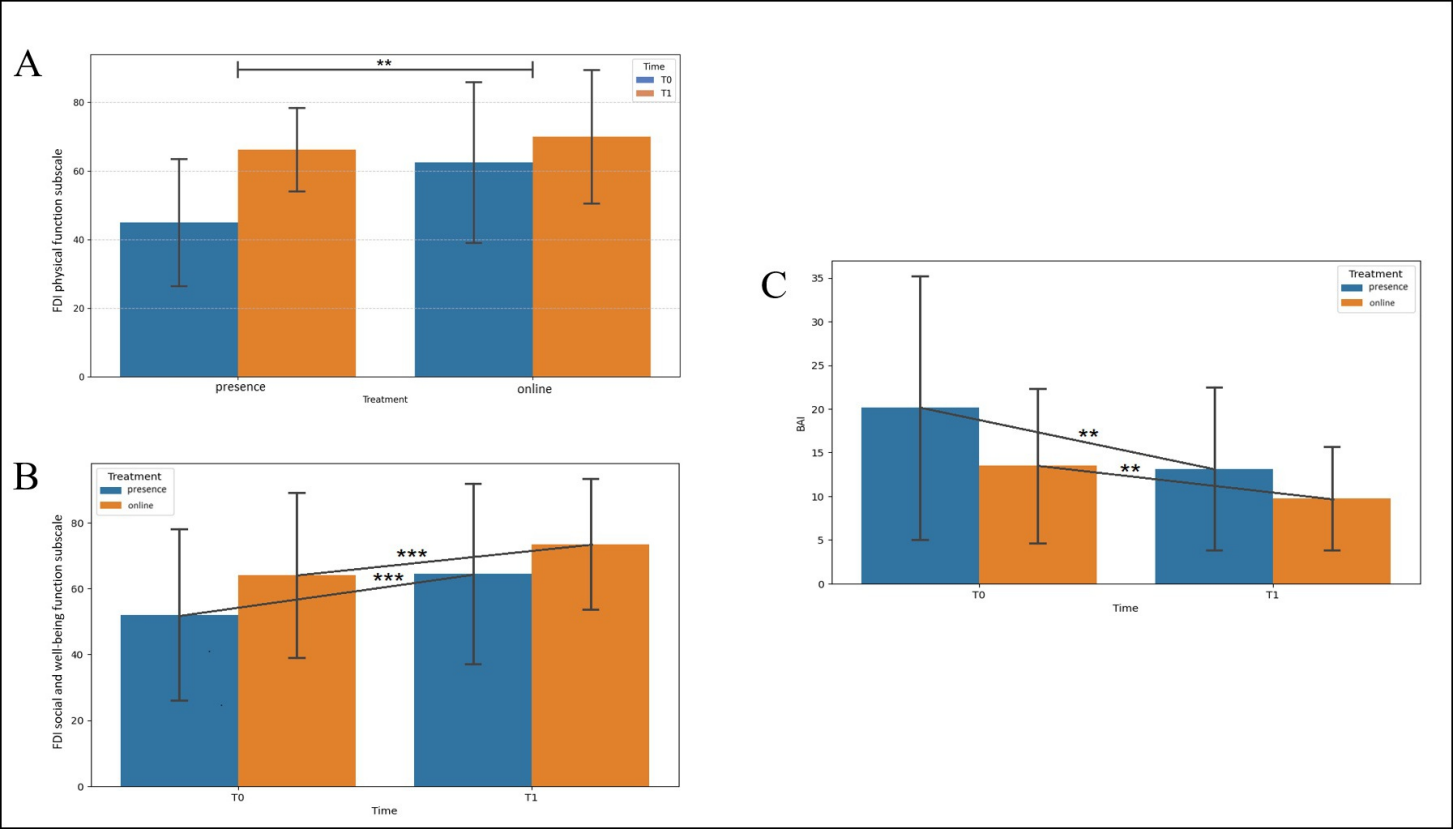
Changes in (A) Facial Disability Index (FDI) physical function subscale, (B) FDI social and well-being function subscale, and (C) Beck Anxiety Inventory (BAI) total score. Data are given as mean and SD. The statistical significance refers to the comparison between in-person and online treatments. **P<.01; ***P<.001 (2-way ANOVA); T0: baseline; T1: posttreatment time point.

**Figure 3.**
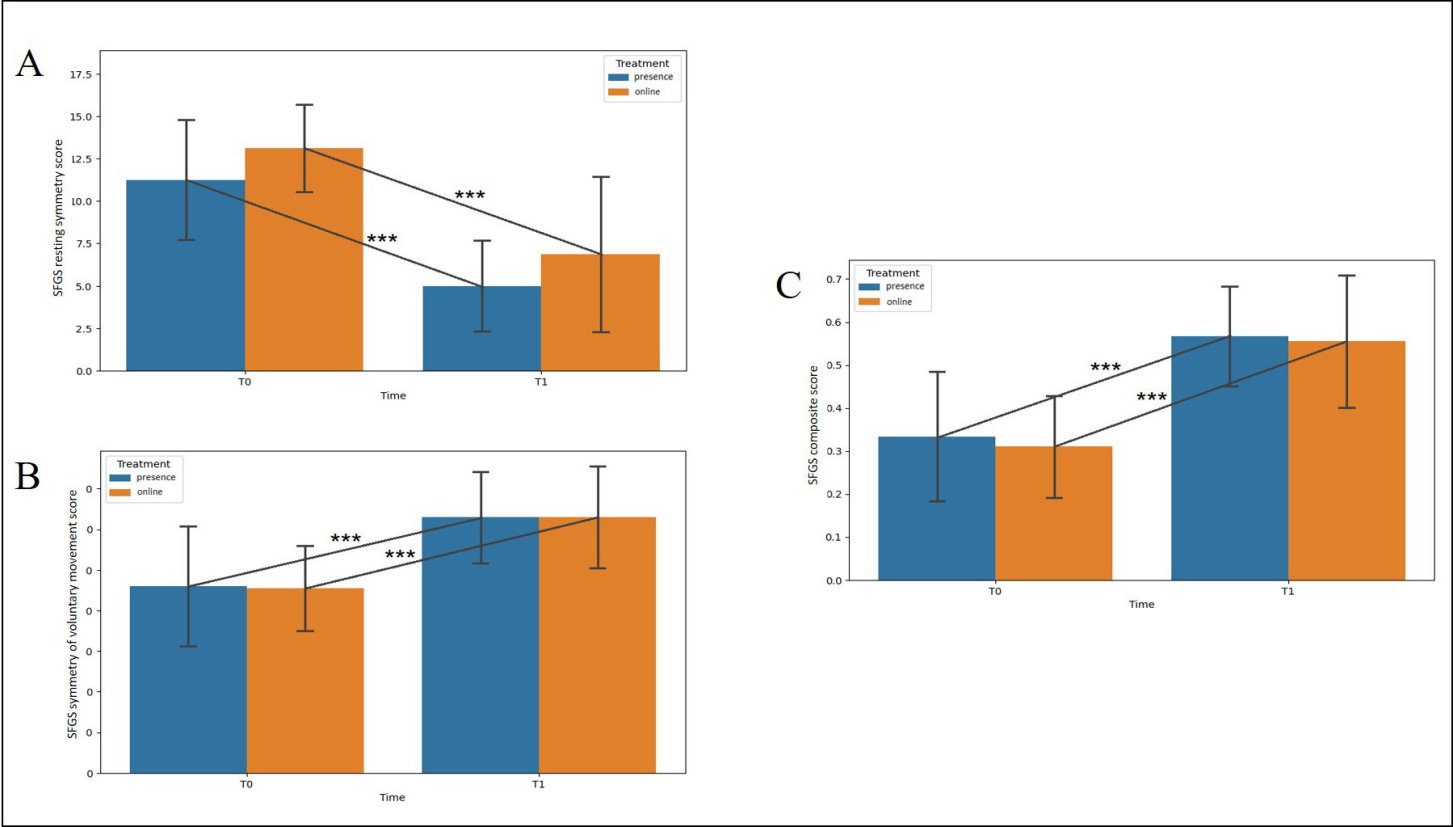
Changes in (A) Sunnybrook Facial Grading System (SFGS) resting symmetry score, (B) SFGS symmetry of voluntary movement score, and (C) SFGS composite score. Data are given as mean and SD. The statistical significance refers to the comparison between in-person and online treatments. ***P<.001 (2-way ANOVA); T0: baseline; T1: posttreatment time point.

**Figure 4.**
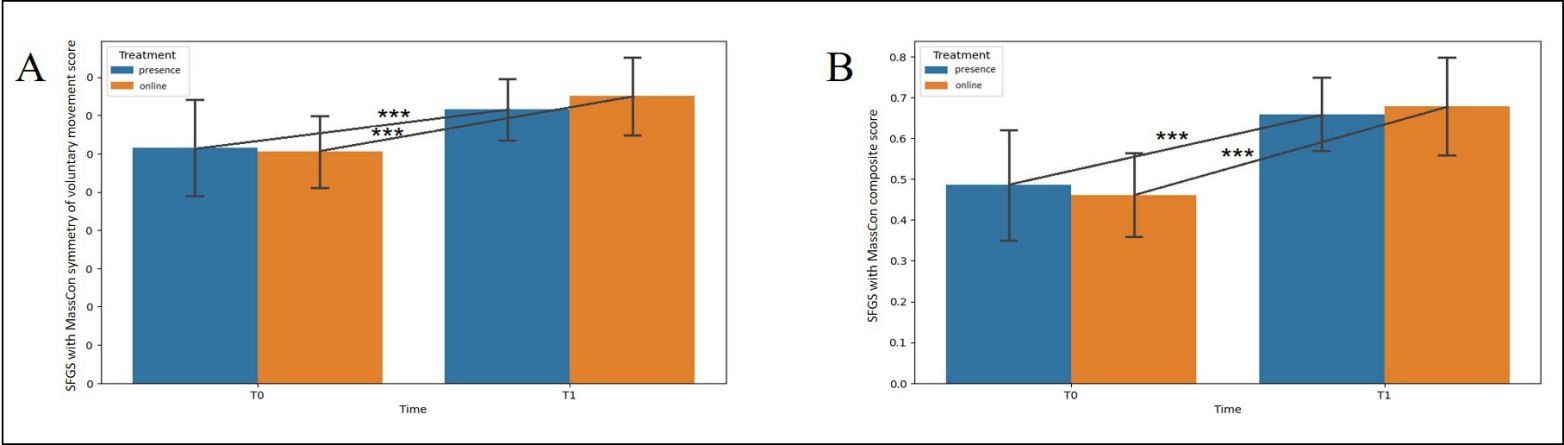
Changes in (A) Sunnybrook Facial Grading System (SFGS) with bilateral masseter contraction (MassCon) symmetry of voluntary movement score and (B) SFGS with MassCon composite score. Data are given as mean and SD. The statistical significance refers to the comparison between in-person and online treatments. ***P<.001 (2-way ANOVA); T0: baseline; T1: posttreatment time point.

Two-way ANOVA results are shown in Table 2. The analysis revealed no significant treatment effect for the outcomes considered (*P*>.05 for all the analyses; Table 2). A time × treatment effect was observed only for the FDI physical function subscale (*F*_1_=14.356; *P*=.002), with post hoc analysis revealing a significant increase between T0 and T1 in the in-person treatment group (mean difference=–21.25, 95% CI −29.125 to −13.375; *P*<.001; Holm-Bonferroni post hoc test; Figure 2A). The effect of time was found for all the outcome variables except for the SFGS synkinesis score (*P*>.05). Specifically, a significant increase in score for the FDI social and well-being function subscale (mean difference=−11, 95% CI −15.163 to −6.837; *F*_1_=32.114; *P*<.001) and decrease in the BAI total score (mean difference=5.375, 95% CI 2.357-8.393; *F*_1_=14.592; *P*=.002; Figures 2B and 2C, respectively) were detected, a significant decrease in the SFGS resting symmetry score (mean difference=6.250, 95% CI 4.354-8.146; *F*_1_=50.00; *P*<.001) and increase in the SFGS symmetry of voluntary movement score (mean difference=−17.250, 95% CI −21.111 to −13.389; *F*_1_=91.810; *P*<.001) and SFGS composite score (mean difference=−0.239, 95% CI −0.288 to −0.191; *F*_1_=111.430; *P*<.001) were observed (Figures 3A-3C, respectively), and a significant increase in the SFGS with MassCon symmetry of voluntary movement score (mean difference=−12.250, 95% CI −15.486 to −9.014; *F*_1_=65.910; *P*<.001) and SFGS with MassCon composite score (mean difference=−0.196, 95% CI −0.231 to −0.161; *F*_1_=143.499; *P*<.001) were observed (Figures 4A and 4B, respectively). No dropouts were registered in either treatment group, indicating strong patient engagement and good adherence to the intervention across both delivery modalities.

**Table 2.** Clinical assessments for each treatment condition (in-person and online) at baseline (T0) and the posttreatment time point (T1) and 2-way ANOVA results.

	In-person, mean (SD)		Online, mean (SD)		2-way ANOVA, *P* value		
	T0	T1	T0	T1	Time	Treatment	Time × treatment
SFGS^a^							
Resting symmetry score	11.25 (3.54)	5 (2.67)	13.13 (2.59)	6.88 (4.58)	*<.001* ^b^	.22	>.99
Symmetry of voluntary movement score	46 (14.81)	63 (11.26)	45.5 (10.46)	63 (12.60)	*<.001*	.97	.89
Synkinesis score	1.38 (2.13)	1.25 (1.49)	1.38 (1.69)	0.63 (1.06)	.32	.66	.47
Composite score (%)	33.4 (15.1)	56.8 (11.6)	31 (11.8)	55.5 (15.4)	*<.001*	.78	.81
SFGS with MassCon^c^							
Symmetry of voluntary movement score	61.5 (12.64)	71.5 (8.12)	60.5 (9.42)	75 (10.2)	*<.001*	.80	.16
Composite score (%)	48.5 (13.6)	65.9 (9)	46.1 (10.2)	67.9 (12)	*<.001*	.97	.20
FDI^d^							
Physical function subscale	45 (18.51)	66.25 (12.17)	62.5 (23.45)	70 (19.46)	*<.001*	.27	*.002*
Social and well-being function subscale	52 (26.01)	64.5 (27.41)	64 (25.02)	73.5 (19.94)	*<.001*	.41	.45
BAI^e^ total score (0-63)	20.13 (15.11)	13.12 (9.34)	13.5 (8.85)	9.75 (5.95)	*.002*	.33	.27

a SFGS: Sunnybrook Facial Grading System.

b Italicized values are significant.

c MassCon: bilateral masseter contraction.

d FDI: Facial Disability Index.

e BAI: Beck Anxiety Inventory.

Finally, correlation analysis revealed a moderate positive correlation between age and change in SFGS with MassCon symmetry of voluntary movement score (*r*=0.50, 95% CI 0.01-0.799; *P*=.05) and a strong positive correlation between the time from surgery to rehabilitation and change in SFGS composite score (*r*=0.94, 95% CI 0.832-0.979; *P*=.005; Table 3).

**Table 3.** Results of correlation analysis through Pearson correlation.

	Age		Onset to surgery		Surgery to rehabilitation		Onset to rehabilitation	
	Pearson *r*	*P* value	Pearson *r*	*P* value	Pearson *r*	*P* value	Pearson *r*	*P* value
ΔSFGS^a^ resting symmetry score^b^	0.05	.87	0.44	.39	−0.75	.09	0.18	.73
ΔSFGS symmetry of voluntary movement score^c^	0.17	.52	−0.37	.47	0.8	.06	−0.1	.85
ΔSFGS synkinesis score	0.10	.70	−0.3	.57	0.046	.93	−0.28	.59
ΔSFGS composite score	0.1	.72	−0.43	.39	*0.94* ^d^	*.005*	−0.11	.83
ΔSFGS with MassCon^e^ symmetry of voluntary movement score	*0.50*	*.05*	−0.31	.54	−0.24	.65	−0.39	.44
ΔSFGS with MassCon composite score	0.34	.19	−0.54	.27	0.25	.64	−0.44	.38
ΔFDI^f^ physical function subscale	0.43	.10	−0.04	.94	−0.36	.48	−0.16	.76
ΔFDI social and well-being function subscale	0.46	.07	−0.4	.44	−0.59	.22	−0.59	.22
ΔBAI^g^ total score	−0.11	.70	0.32	.54	0.55	.26	0.49	.32

a SFGS: Sunnybrook Facial Grading System.

b Variation of outcome scores, calculated with the following formula: Δ=variation of outcome scores, as for the formula: ∆ score=T1 score−T0 score.

c Posttreatment time point (T1) score − baseline (T0) score.

d Italicized values are significant.

e MassCon: bilateral masseter contraction.

f FDI: Facial Disability Index.

g BAI: Beck Anxiety Inventory.

## Discussion

### Principal Findings

Despite the pivotal importance of PT, the most effective PT approach after surgically treated peripheral FNP is still unclear, with a telerehabilitation-based emotional training protocol showing promising foundations given the specific characteristics of the facial impairments. In this exploratory pilot study, we confirmed the feasibility and potential clinical value of a telerehabilitation-based emotional training protocol in improving motor symptoms, facial disability, and anxiety in patients with peripheral FNP treated with triple innervation surgery also when compared with an in-person approach. However, these findings should be considered preliminary.

Emotional training is a novel therapeutic approach based on multiple phases that has shown preliminary promising results in the motor rehabilitation of FNP [30]. The first phase comprises a sensory-based intervention in which patients are asked to recognize the sensations induced by external stimuli on the lesioned hemiface (eg, intrinsic characteristics of objects or surfaces and passive movements performed by the physiotherapist) and perform facial movements—or imagine them, given that both motor imagery and actual movements activate the same neural circuits [48,49]. The rationale behind this first phase relies on the theories of motor control [55] that conceptualize the normal control of movement as a cognitive task [32] in which individuals integrate sensory inputs (feedback control), prior knowledge, and volitional intention (feedforward control) to select appropriate motor programs and regulate motor output [56,57]. As reported by numerous studies, sensory stimulation reshapes cortical motor representations [58] given the interplay between motor (primary motor cortex, supplementary motor area, and premotor cortex) and somatosensory (primary and secondary somatosensory cortices and the sensory thalamus) regions [59,60]. In neurological motor rehabilitation, sensory-based interventions have been shown to enhance adaptive motor cortical plasticity and promote functional recovery [61-63]. Nevertheless, cognitive processes (eg, information perception and the elaboration, selection, and execution of motor plans) [31,64] play a critical role in mediating these mechanisms, supporting functional interactions between the body and the environment [65,66]. In addition, combining different types of sensory information stimulates the activity of associative brain areas that are important for learning, neuronal plasticity, and recovery [67]. Although evidence remains limited [64,68-71], promising findings suggest that sensory-based interventions can significantly improve the ability to perform activities of daily living and enhance quality of life [71] even for people with peripheral FNP [72,73].

The second phase aims to restore authentic facial expressions, emotional congruence, emotional symmetry, and spontaneity [74], which is crucial for optimal motor recovery [75]. At the neurophysiological level, facial movements in response to emotions are mediated by a widespread neural network involving various cortical regions that project to the facial nucleus, integrating motor planning, somatosensory feedback, and limbic system activity [76,77]. While corticofacial projections from the lateral frontal lobe contribute to the voluntary control of facial movements [24], the cingulate cortex plays a pivotal role in emotional expression through facial mimics, with projections from the amygdala providing the necessary emotional context [24]. For example, among the 2 different neural pathways controlling laughter [25], one, which involves primarily the frontal operculum [78,79] and the primary motor cortex, represents the neural basis of voluntary, nonemotional, conversational laughter; the other, which includes the pregenual anterior cingulate [80,81], ventral striatum and nucleus accumbens, amygdala, and hypothalamus [82], is involved in the production of emotional laughter, with the supplementary motor area likely modulating the production of both [27]. Notably, training voluntary movements to express different emotions helps reconnect the limbic-motor pathways [24], supporting the synergy between voluntary and emotional facial movements [24]. This is particularly important for facial nerve lesions, where voluntary movement and emotional expression are often dissociated [83]. The final phase of rehabilitation focuses on functional exercises designed to restore the ability to perform essential daily activities. These functional tasks, such as eating, speaking clearly, or demonstrating facial expressiveness during conversations, involve complex and coordinated facial muscle activity [84]—which is why these exercises are included as the final part of the treatment. By training patients in real-life contexts, they are engaged in meaningful activities that mirror their daily life. This approach has been shown to enhance neuroplasticity and functional recovery through task-specific repetition [85]—a principle known as “use-dependent cortical reorganization” [86]. In addition, focusing on real-world tasks aligns rehabilitation with the patient’s meaningful goals, improving motivation and adherence to the treatment protocol [87-89].

Taken together, our findings reinforce the conclusions of several systematic reviews on the feasibility of telerehabilitation in neurological conditions [33,90]. Recently, a randomized controlled trial investigated the effectiveness of video exercise–based telerehabilitation on motor and psychological symptoms in patients with peripheral FNP [35]. Patients in the telerehabilitation group not only found the treatment satisfactory and accessible but also demonstrated a significant improvement in FDI scores comparable to those receiving routine in-person care. Furthermore, both groups exhibited similar improvements in anxiety after treatment [35]. Despite the limitations of our study design, our findings are consistent with this evidence, suggesting the need to expand upon the value of telerehabilitation services for peripheral FNP, also considering that both patients and therapists generally have a positive attitude toward the integration of digital technologies [91,92]. Following the telerehabilitation-based emotional training, our patients reported improvements comparable to those of the face-to-face treatment in facial nerve function and facial symmetries (both at rest and during voluntary movement) even when considering the modified version of the SFGS (SFGS with MassCon). This is consistent with the only study assessing the effectiveness of emotional training in peripheral FNP, although the latter was performed in an in-person fashion [30]. Indeed, the authors reported that 50 patients who underwent face-to-face emotional training had improved House-Brackmann scale scores and symmetry of the face both at rest and during movements. In addition, in contrast to our study, only patients with iatrogenic peripheral FNP but not those who underwent surgery had gains in social function and subjectively perceived well-being. In our study, all patients experienced significant improvements in social and well-being function at the end of the treatment both in the in-person and online groups. This result was further confirmed by improved anxiety assessments. This is of particular importance given the substantial psychosocial burden that patients with facial impairments experience [93]. Nevertheless, the nonrandomized allocation and limited sample size warrant a cautious interpretation of these positive findings. Moreover, comparisons should be interpreted cautiously as the aforementioned study [30] was a retrospective analysis with no control group and the authors performed an in-person treatment protocol. However, in this study, self-reported physical function was greater at the end of the in-person treatment when compared to telerehabilitation treatment. At least for physical functioning, the therapeutic alliance and the therapist-patient relationship, which are strengthened by the direct, in-person interaction, and the emotional bond inherent to in-person rehabilitation may have had a positive effect, as previously suggested [33,94].

This study has some limitations. First, the small sample size may have underpowered the study and increased the risk of inflated effect estimates (eg, from correlations), whereas the use of subjective and operator-dependent assessments might have affected the results and interpretation. The nonrandomized allocation could have affected the internal validity of the study, whereas the absence of a follow-up assessment prevents conclusions regarding the durability of the observed improvements. In addition, our neurophysiological hypotheses should be confirmed through neuroimaging studies, which were not feasible at this time. Second, although our patients were asked to maintain a stable pharmacological treatment throughout the study, the pharmacological therapies of the patients were not documented. The different biochemical effects induced by the medications could have influenced motor recovery, as previously suggested [95,96]. Similarly, we included surgically treated patients with peripheral FNP with multiple palsy etiologies (eg, neurinoma and trauma). The different underlying mechanisms of facial dysfunction might have affected the results and reduced the external validity of our findings.

### Clinical Implications

The findings of this pilot study indicate that emotional training could be integrated into routine postoperative care for patients with peripheral FNP after triple innervation surgery, offering measurable benefits in facial symmetry, voluntary motor performance, social functioning, and anxiety reduction. The intervention’s emphasis on both motor and psychosocial domains aligns well with the complex functional demands of patients undergoing facial reanimation, who often experience challenges that extend beyond motor impairment alone. Interestingly, the comparable improvements observed with the in-person protocol and telerehabilitation delivery suggest that emotional training can be implemented flexibly without compromising clinical outcomes, providing a viable option for individuals with mobility limitations, work constraints, or psychosocial discomfort associated with facial impairment. The structured nature of emotional training, centered on restoring selective motor control, improving emotional expression, and reducing maladaptive compensatory patterns, offers clinicians a reproducible framework that can be tailored to individual patient needs. Clinicians may consider telerehabilitation as a practical strategy to maintain continuity of care and reduce barriers to treatment access.

### Conclusions

In conclusion, this exploratory pilot study provides preliminary evidence that telerehabilitation-based emotional training may have beneficial effects on motor, physical, and psychosocial functioning, as well as on anxiety, in surgically treated patients with peripheral FNP. Given the study’s observational design and limited sample size, these findings should be interpreted cautiously and considered hypothesis generating rather than confirmatory. Further studies with optimized designs (eg, double-blind, parallel-group trials), larger sample sizes, and more objective and informative assessments (such as neurophysiological or neuroimaging evaluations) are warranted to confirm and extend these findings.

## Supplementary material

10.2196/79520 Multimedia Appendix 1 Detailed, structured session plan with protocol phases, specific exercises, session organization, approximate durations, and progression criteria.

## Abbreviations

BAI: Beck Anxiety Inventory
CONSORT: Consolidated Standards of Reporting Trials
FDI: Facial Disability Index
FNP: facial nerve palsy
PT: physiotherapy
SFGS: Sunnybrook Facial Grading System
SFGS with MassCon: Sunnybrook Facial Grading System with bilateral masseter contraction
