# Supplementary material for: Emotional Training via Telerehabilitation After Surgical Treatment for Facial Palsy: Prospective, Assessor-Blinded, 2-Arm Pilot Cohort Study
Source: JMIR Rehabil Assist Technol. 2026 Apr 27;13:e79520. doi: 10.2196/79520 (PMC13118142; doi:10.2196/79520)
Supplement: Multimedia Appendix 1 [file rehab-v13-e79520-s001.pdf]

Table SI. Structured session plan for ET treatment

PHASE	OBJECTIVES	ACTIVITIES / EXERCISES	DURATION PER SESSION	PROGRESSION CRITERIA
Manual Therapy	I) Mobilize and lengthen soft tissues;<br>II) Improve tissue flexibility.	Intraoral and extraoral stretching techniques and transverse massage targeting the masseter, pterygoid and zygomaticus muscles, orbicularis oculi and orbicularis oris of the palsy hemiface	ca. 10 min	I) Improved tissue flexibility on palpation and mobilization;<br>II) Enhanced symmetry during functional and emotional expressions;<br>III) Reduced patient-reported stiffness or discomfort during facial movements.
Neurocognitive Sensory Training	I) Enhance sensory discrimination from the palsy hemiface;<br>II) Improve proprioceptive feedback from the palsy hemiface;<br>III) Optimizing the use of sensory information to plan movement in the palsy hemiface.	I) Tactile discrimination task: the patient distinguishes lengths of graded tactile bars of different lengths (1–4 cm) placed on the palsy hemiface, with and without vision;<br>II) Bilateral comparison task: the patient compares the tactile stimuli between hemifaces (palsy vs. healthy);<br>III) Oral proprioception task: patient identifies specific characteristics (height, texture) of bars of different thickness and consistency positioned between the teeth by interpreting the cheek movements done while teeth clenching;<br>IV) Left-Right comparison: the patient closes the eyes and/or smiles, while clenching the teeth, and compare right–left differences in eyelid/lips contact, movement speed, and end-position sensation;<br><br>As the patient progresses, the same task is performed without teeth clenching.	ca. 15 min	I) Ability to reliably discriminate stimuli;<br>II) Improved symmetry in sensory perception;<br>III) Reduction in compensatory masseter overactivation.
Emotional Expression Training	I) Restore emotional congruence, symmetry, and spontaneity of facial expressions.	I) Real-life emotional movements: the patient produces smiles of three different amplitudes while clenching the teeth, each associated with a distinct real-life emotional scenario. In each expression, the patient must consistently maintain right–left symmetry;	ca. 15 min	I) Improved ability to produce symmetric expressions;<br>II) Reduced dissociation between voluntary and emotional expression;

		II) Memory comparison: the patient re-evokes and expresses the emotion defined by Osgood (happiness, sadness, interest, disgust, fear, anger, and surprise) while clenching the teeth, monitoring facial symmetry and overall movement, and comparing the current performance with the remembered expression;<br>III) Replication of emotions: the patient imitates, while clenching the teeth, the emotions defined by Osgood (happiness, sadness, interest, disgust, fear, anger, and surprise) presented by the therapist using drawings, photographs, or videos. As the patient improves, he/she is asked to switch from an emotion to another.<br><br>As the patient progresses, the same tasks are performed without teeth clenching.		III) Transition from clenching-assisted activation to independent facial nerve activation.
Functional Facial Tasks	I) Restore facial function during everyday activities like communication, articulation, mastication, drinking.	I) Articulation exercises: the patient reads aloud a short text or a list of words containing labial sounds. While reading, the patient focuses on producing clear sounds, moving the lips symmetrically, and maintaining control of the facial muscles.<br>II) Mastication exercises: the patient practices suck a liquid through a straw and chewing slowly and evenly on both sides of the mouth. The patient then transfers small amounts of food or liquid from one cheek to the other, paying attention to smooth movement and avoiding compensations.	ca. 10 min	I) Enhanced symmetry and coordination of lip and cheek movements during functional tasks.<br>II) Improved clarity and precision of speech during articulation tasks, with reduced distortions of labial sounds.<br>III) Ability to chew or transfer food or liquid bilaterally without compensatory movements, such as jaw deviation or excessive masseter activation.
